# Clinical database of the CYP-guides trial: An open data resource on psychiatric hospitalization for severe depression

**DOI:** 10.1016/j.dib.2020.105457

**Published:** 2020-03-20

**Authors:** Joseph Tortora, Saskia Robinson, Seth Baker, Gualberto Ruaño

**Affiliations:** a Research Administration, Hartford Hospital, 80 Seymour Street, Hartford, CT 06102, United States; b Information Technology Services, Hartford Healthcare, 5 Batterson Park Road, Farmington, CT 06032, United States; c Institute of Living at Hartford Hospital, Genomas Laboratory of Personalized Health, 67 Jefferson Street, Hartford, CT 06106, United States

**Keywords:** Major depressive disorder, Depression, Psychotropic utilization, Hospital length of stay, Hospital readmission rate, Clinical decision support, Personalized medicine

## Abstract

CYP-GUIDES *(Cytochrome Psychotropic Genotyping Under Investigation for Decision Support****)*** is a randomized controlled trial (RCT) of pharmacogenetic decision support in the psychotropic treatment of hospitalized patients with major depressive disorder or severe depression. Patients were treated according to their CYP2D6 functional status or empirically and compared for both their *Length of Stay* (LOS, primary outcome) at the hospital and *Re-Admission Rate* (RAR, secondary outcome) 30 days after discharge.

The trial was conducted at the Institute of Living (Hartford Hospital, Hartford CT). *CYP2D6* genotyping was implemented to infer the functional status of the CYP2D6 enzyme and classify it as sub-normal, normal or supra-normal. The electronic medical record (EMR) transmitted to the physician the indicated drug prescribing guidance.

During the RCT, 1500 patients were recruited and 1459 genotyped for *CYP2D6*. A 1:2 randomization assigned 477 patients to standard therapy (*Group S*) and 982 to genetically-guided therapy (*Group G*). In *Group S,* standard empiric treatment was indicated for all patients. In *Group G*, medications primarily metabolized by the CYP2D6 enzyme were proscribed for patients with sub- or supra-normal CYP2D6 function.

The clinical course, therapeutic guidance, and drug treatment for each patient are being published in this article and deposited in *Mendeley Data*. These data should be valuable to assess the impact of clinical decision support on utilization of psychiatric resources for treatment of severe depression requiring hospitalization.

Specifications Table

**Table:** 

Subject	Psychiatric hospitalization and severe depression
Specific subject area	Psychotropic medication administration to inpatients with major depressive disorder or severe depression at a psychiatric hospital
Type of data	Table Chart
How data were acquired	Data were acquired directly from servers for the electronic medical records and by manual review of patient medical charts.
Data format	Raw Analyzed Filtered
Parameters for data collection	Therapeutic guidance charts were obtained from the CYP-GUIDES online Portal. For patient randomization, ISAAC, a 64 bit shift MD5 program, was used. All other data were extracted from the electronic medical record.
Description of data collection	Potential participants were screened based on inclusion/exclusion criteria. Those who met criteria were then approached and consented in person. Data for enrolled patients were then imported from the electronic medical record upon entry into a trial-specific database. Any data not imported were manually entered. Therapeutic guidance charts were provided to physicians according to each patient's group assignment and CYP2D6 functional status obtained from a separate *CYP2D6* genotype database.
Data source location	Hartford, CT United States 41.7496353,−72.6834961
Data accessibility	Repository name: *Mendeley Data* Data identification number: *10.17632/25yjwbphn4.1* Direct URL to data: https://data.mendeley.com/datasets/25yjwbphn4/1
Related research article	Ruaño G, Holford T, Seip RL, Goethe JW, Mehendru R. Pharmacogenetic clinical decision support for psychiatric hospitalization: Design of the CYP-GUIDES randomized controlled trial. *Contemporary clinical trials*. 2019; 83:27–36. [PMID: 31247284] Ruaño G, Robinson S, Holford T, Mehendru R, Baker S, Tortora J, Goethe JW. Results of the CYP-GUIDES Randomized Controlled Trial: Total Cohort and Primary Endpoints. *Contemporary clinical trials*. 2020 Feb 01; 89:05910. [PMID: 31838256]

## 1 Value of the data

• This database describes the clinical course of 1500 inpatients with major depressive disorder or severe depression at a tertiary psychiatric hospital.
• Clinical investigators, healthcare administrators and actuaries will be able to assess resource utilization during psychiatric hospitalization.
• The database could be analyzed for covariates and specific sub-cohorts defined by ethnicity, drug prescription or length of stay.
• Drug prescription patterns are well documented based on number of different psychotropics as well as total number of administrations.
• Identifiers are provided for the psychiatrists treating the patients, which could enable evaluation of physician behavior.
• The specific therapeutic guidance provided for each patient is identified to support trial on the use of clinical decision support in psychiatric hospitalization.

## 2 Data description

The dataset in this article provides basic demographic characteristics, along with information regarding trial group assignment, length of stay, 30-day readmission, and medication administration during the hospital stay.

Data have been de-identified in accordance with the standards of the Privacy Rule of the Health Insurance Portability and Accountability Act (HIPAA), utilizing the Safe Harbor method [1]. In accordance with this method, the data do not contain the 18 personal identifiers specified, and no subjects are included with an age above 89. A unique code (ID) has been assigned to each subject as specified by the de-identification implementation standards. These data are longer considered individually identifiable health information as there is no reasonable basis to believe that the information can be used to identify an individual.

Data in the total dataset are described as follows:

**ID** – Unique identification number assigned to each patient who was enrolled in the trial.
**GENDER** – Male or female.
**AGE** – The age, in years, of each patient at the time of enrollment.
**RACE/ETHNICITY** – Race was self-reported by the patient from a system database, which included “White”, “Black”, “Latino”, and “Other/Unknown” as options. Therefore, this column is referred to as “Race/Ethnicity”.
**DIAGNOSIS** – The diagnosis given upon admission to the hospital, which was used by the trial coordinator to evaluate each patient for inclusion in the trial.
**MD** – Alphabetic code assigned to each hospital physician who cared for the patients in the trial.
**ASSIGNMENT** – Patients were randomly assigned to *Group S* or *Group G* in a *1:2* ratio. Patients in *Group S* received standard care, whereas patients in *Group G* had their psychotropic prescriptions guided by their CYP2D6 functional status.
**ELECTRONIC MEDICAL RECORD (EMR)** – During the course of the trial, 2 platforms for EMR were employed: the Clinical Evaluation and Monitoring System *(C)* and the Epic^Ⓡ^ *(E)* EMR. The first 856 patients were recruited and followed under CEMS, and the subsequent 644 under Epic^Ⓡ^.
**LOS** – Length of Stay (in hours) at the psychiatric hospital (IOL), defined and calculated as the date/time of discharge minus the date/time of admission.
**RAR** – 30 day *Re*-Admission Rate. A “1” denotes that the patient was readmitted at least once during the thirty days after they were discharged. A “0” denotes no evidence of a readmission during the same time period could be found.
**COLUMNS** *A* through *AD* – Coded columns for the medications and the number of administrations for each during the hospital stay. Each Column represents a different medication from the formulary as prescribed and comprised of the following drugs: Amitriptyline, Aripiprazole, Asenapine, Bupropion, Chlorpromazine, Citalopram, Clomipramine, Clonidine, Doxepin, Duloxetine, Escitalopram, Fluoxetine, Fluphenazine, Fluvoxamine, Guanfacine, Haloperidol, Imipramine, Lithium, Methylphenidate, Mirtazapine, Nortriptyline, Olanzapine, Paliperidone, Perphenazine, Quetiapine, Risperidone, Sertraline, Trazodone, Venlafaxine, Ziprasidone. A key for these columns is included in the dataset. Each number corresponds to the number of times that the given medication was administered to the patient while in the hospital.
**# PSYCHOTROPIC MEDICATIONS** – The number of distinct psychotropic medications each patient received during the participant's hospital stay. For example, if a patient received Aripiprazole (2 administrations), Fluoxetine (3 administrations), and Trazodone (4 administrations), the number of different psychotropic medications administered would be 3.
**# ADMINISTRATIONS** – The count of the times a given psychotropic medication was administered to each patient during the participant's hospital stay. In the example above, the 2 administrations of Aripiprazole, 3 administrations of Fluoxetine, and 4 administrations of Trazodone, would be counted and reported in the respective column for the specific drug for each patient.
**THERAPEUTIC GUIDANCES** – There were 3 guidance charts where the psychotropic drugs were colorized according to the patient's group assignment and CYP2D6 functional status: *(a) Evergreen (EG)*, where all drugs are colorized green and can be prescribed as usual; *(b) Christmas Tree (CT)*, where CYP2D6 major substrate drugs are colorized red, indicating proscription, and the others green; and *(c) Traffic Light (TL)*, where CYP2D6 minor substrate drugs are also colorized yellow, indicating caution. In the case of *Group S*, the guidance provided for all patients was the *Evergreen* drug chart, regardless of the patient's CYP2D6 functional status. In the case of *Group G*, drug chart choices among the 3 options depended on the patient's CYP2D6 function.
**REPRESENTATIVE DATA** – The dataset has been deposited with *Mendeley Data* [2]. The following represent descriptions of cohort characteristics and illustrative analysis enabled by the dataset.

*Demographics.* Demographics for the total cohort and database of the CYP-GUIDES trial are shown in Table 1.

**Table 1:** Demographics by gender, age, and race/ethnicity for the total cohort in the database.

Gender	Age Group	White	Black	Latino	Other/Unknown	Total
Female	18-20	55	21	37	12	125 (16.3%)
	21-30	116	30	42	16	204 (26.5%)
	31-40	58	16	41	3	118 (15.3%)
	41-50	80	21	36	6	143 (18.6%)
	51-60	64	14	32	2	112 (14.6%)
	>60	51	3	12	1	67 (08.7%)
Total Females		424 (55.1%)	105 (13.7%)	200 (26.0%)	40 (05.2%)	769 (51.3%)
Male	18-20	47	8	11	7	73 (10.0%)
	21-30	104	17	51	8	180 (24.6%)
	31-40	65	13	26	5	109 (14.9%)
	41-50	78	15	53	6	152 (20.8%)
	51-60	95	21	32	4	152 (20.8%)
	>60	51	3	9	2	65 (08.9%)
Total Males		440 (60.2%)	77 (10.5%)	182 (24.9%)	32 (04.4%)	731 (48.7%)
*Grand Totals*		*864 (57.6%)*	*182 (12.1%)*	*382 (25.6%)*	*72 (04.8%)*	*1500*

**Fig. 1:**
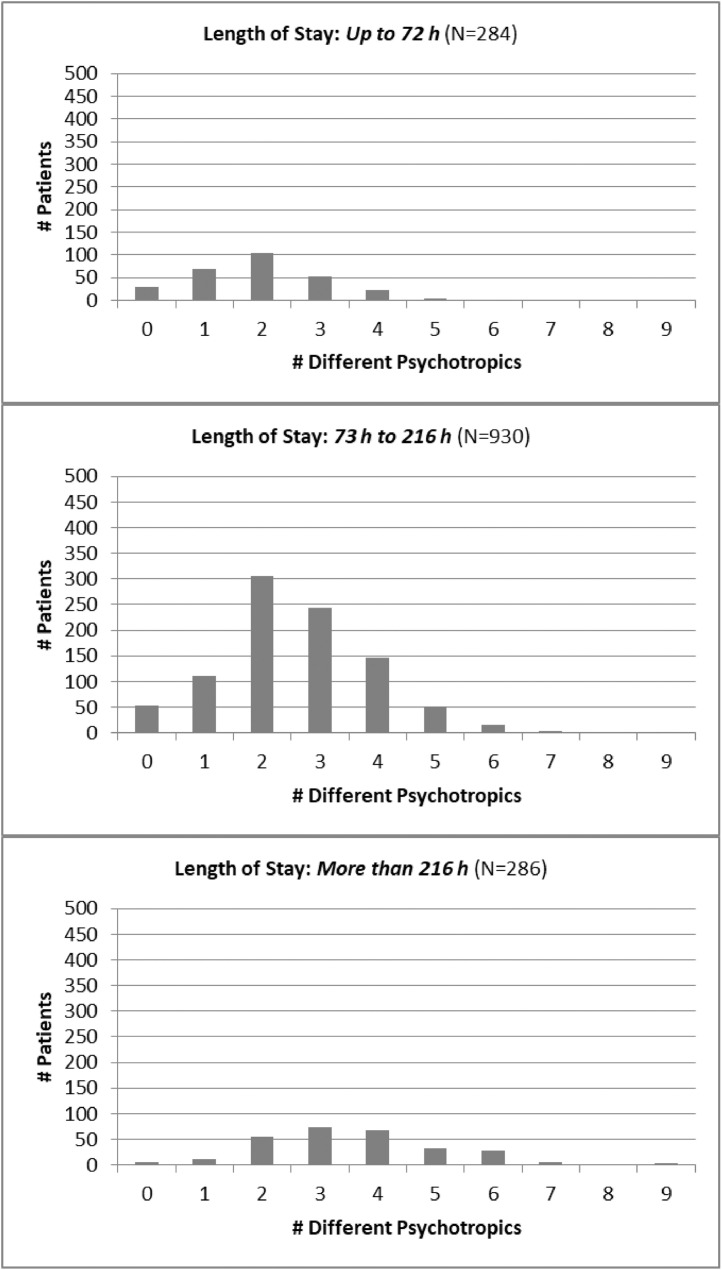
Psychotropic drug selections in relationship to Length ot Stay. Each bar represents the number of patients who received that many different psychotropic medications during hospitalization.

**Fig. 2:**
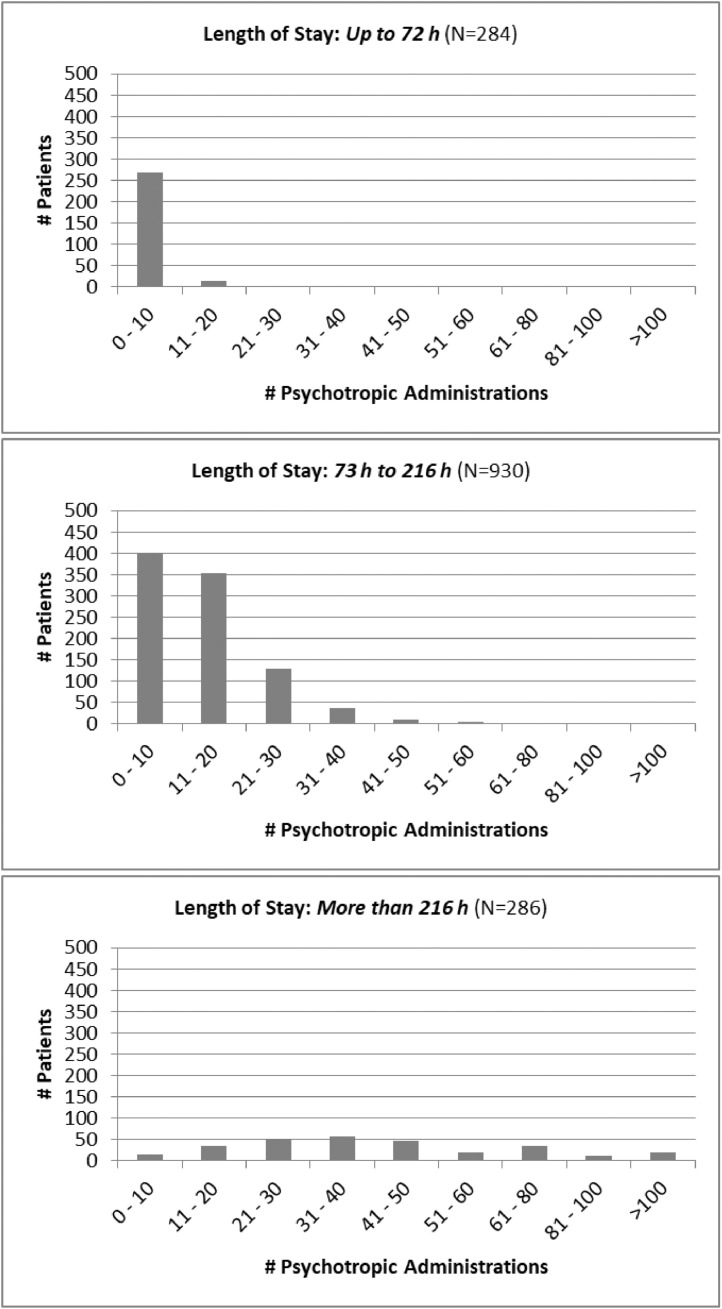
Psychotropic drug administrations in relationship to Length of Stay. Each bar represents the number of patients who received that many administrations of all psychotropic drugs during hospitalization.

*Drug utilization data.* Examples of the use of the data for drug utilization research are shown in Figs. 1 and 2. The stratification in these figures is by Length of Stay, the primary outcome, to assess the dependence of drug utilization with hospitalization. LOS was stratified into 3 time intervals: LOS of 72 h or less (3 days maximum) *(top panel)*, LOS between 73 h and 216 h (9 days maximum) *(middle panel)*, and LOS of more than 216 h *(bottom panel)*. Fig. 1 describes the number of different psychotropic drugs administered whereas Fig. 2 describes the total number of psychotropic administrations during hospitalization.

## 3 Experimental design, materials, and methods

### 3.1 Trial design

In the CYP-GUIDES study, a prospective RCT, outcomes in patients with major depressive disorder or severe depression were compared among those treated according to CYP2D6 functional status versus those treated following standard, empiric psychotropic selection.

Methodology for the trial has been detailed in a published report [3], and the findings have been reported for the total cohort [4]. The RCT was registered in *ClinicalTrials.gov* as *Pharmacogenetic Decision Support IT System for Psychiatric Hospitalization: RCT (CYP-GUIDES)* and assigned identifier NCT 02120729 in the registry [5].

#### 3.1.1 Trial site

The Institute of Living (IOL) at Hartford Hospital was the single site where the RCT was conducted. A clinical coordinator trained by the psychiatric nursing staff and by the investigators consented all patients. A self-identified ethnicity was provided by each patient at admission. *CYP2D6* genotyping and functional phenotyping was performed onsite at Hartford Hospital by the clinical Laboratory of Personalized Health (LPH).

#### 3.1.2 Inclusion/Exclusion criteria

The following were criteria for Inclusion: *(a)* age 18 y or older, (b) all men or women not pregnant or nursing, *(c)* admission to the Institute of Living for major depressive disorder or severe depression [6], *(d)* capacity to understand and comply with the trial's requirements, procedures and protocol. The following were criteria for Exclusion: *(a)* all children and adolescents, *(b)* admission to the hospital 30 days prior to current admission, *(c)* diagnoses or history of dementia, Alzheimer's disease, chronic kidney disease, hemorrhagic stroke, subarachnoid hemorrhage, ischemic stroke or surgery within 6 weeks, *(d)* current participation in any other clinical trial for investigational drugs or devices.

#### 3.1.3 Protection of human subjects

Informed consent was developed and approved by the Hartford Hospital Institutional Review Board (Protocol RUAN004090HE). The informed consent listed and explained to patients the risks and benefits of their participation in the trial. A Data Safety Monitoring Board (DSMB) was established to monitor safety and efficacy of the intervention among all participants and to review data or request statistical analyses as needed for its recommendations.

#### 3.1.4 Randomization

We randomized assignment to *Group S* (Standard care) or *Group G* (Genetically guided) independently of *CYP2D6* genotype. ISAAC, a 64 bit shift MD5 program frequently used for encryption, was adapted for the random assignment. Both patient and physician were blinded to *Group S* versus *Group G* assignment.

#### 3.1.5 Electronic medical record (EMR)

Two platforms for EMR were employed during the course of the study. *Clinical Evaluation and Monitoring System (CEMS)* was embedded within the Behavioral Health Care Information System for 25 years at IOL [7,8] and used from April 2014 to 19 August 2016 for 856 patients. The *Epic*^Ⓡ^ EMR was deployed at IOL as part of organization-wide medical informatics integration by the Hartford Healthcare Corporation and employed thereafter for the subsequent 644 patients.

#### 3.1.6 Outcomes

*Length of Stay* (LOS) [9,10] was the primary outcome. *Re-Admission Rate* (RAR) 30 days after discharge [11,12] was the secondary outcome. Exact calculation of LOS in hours for each patient was enabled by the EMR's timestamps for admission and discharge. To obtain RAR, the EMR was searched for readmissions to the IOL in the 30-day post-discharge period.

### 3.2 Interventions

#### 3.2.1 Genetic information

The Metabolic Reserve (MR) index was developed in 2011 by our group from a study of 1199 psychiatric referrals to derive a quantitative, composite CYP450 functional phenotype [13]. It was then adapted to *CYP2D6* only and validated in a preliminary clinical study of 149 psychiatric inpatients in 2013 [14]. MR is calculated by adding the functional score of each of the 2 *CYP2D6* alleles for each patient.

MR was then implemented when the CYP-GUIDES RCT began in 2014 to derive the CYP2D6 enzymatic function from the patient's *CYP2D6* diplotype. MR values were grouped to index a patient's CYP2D6 functional phenotype to 3 categories: *(a) Sub-functional* (MR 0.0, 0.5 or 1.0), *(b) Functional* (MR 1.5, 2.0 or 2.5), and *(c) Supra-functional* (MR 3.0 or 3.5).

#### 3.2.2 Therapeutic guidances

Physicians were blinded to CYP2D6 functional and to *Group S* versus *Group G* assignment. Instead, drug guidance charts were provided to physicians for the antidepressants, antipsychotics, and stimulants commonly used at IOL.

There were 3 drug guidance charts [3] with all drugs colorized according to their suitability for the patient's CYP2D6 functional status: *(a) Evergreen,* with all drugs colorized green, advising prescription as usual; (*b) Christmas Tree*, with CYP2D6 major substrate drugs colorized red, alerting proscription, and the others colorized green; and *(c) Traffic Light,* with CYP2D6 minor substrate drugs also colorized yellow, alerting caution.

*Evergreen* was indicated for all patients in *Group S. Evergreen* was also indicated for patients in *Group G* with normal CYP2D6 function (MR 1.5, 2.0 or 2.5). *Christmas Tree* was indicated for patients in *Group G* with abnormal CYP2D6 function (MR 0.5, 1.0 or 3.0). *Traffic Light* was indicated for patients in *Group G* with extreme CYP2D6 function (MR 0.0 or 3.5).

## CRediT authorship contribution statement

**Joseph Tortora:** Data curation, Formal analysis, Writing - original draft, Visualization. **Saskia Robinson:** Validation, Investigation, Resources. **Seth Baker:** Software. **Gualberto Ruaño:** Conceptualization, Methodology, Writing - review & editing, Supervision, Project administration, Funding acquisition.

## Appendix Supplementary materials

Image, application 1
